# Hypoxemia in thoracoscopic lung resection surgery using a video double-lumen tube versus a conventional double-lumen tube: A propensity score-matched analysis

**DOI:** 10.3389/fsurg.2023.1090233

**Published:** 2023-02-17

**Authors:** Wei Wang, Zhihao Gong, Mingye Zhao, Zuojing Zhang, Yuwei Qiu, Qiliang Jiang, Jingxiang Wu

**Affiliations:** Department of Anesthesiology, Shanghai Chest Hospital, Shanghai Jiao Tong University, School of Medicine, Shanghai, China

**Keywords:** hypoxemia, double-lumen tube (DLT), video double-lumen tube (VDLT), one-lung ventilation (OLV), thoracoscopic lung resection surgery

## Abstract

**Background:**

Malposition of the double-lumen tubes (DLTs) may lead to hypoxemia during one-lung ventilation (OLV). Video double-lumen tubes (VDLTs) enable continuous observation of DLT position and avoid displacement. We aimed to investigate whether VDLTs could reduce the incidence of hypoxemia during OLV compared with conventional double-lumen tubes (cDLT) in thoracoscopic lung resection surgery.

**Methods:**

This was a retrospective cohort study. Adult patients who underwent elective thoracoscopic lung resection surgery and required VDLTs or cDLTs for OLV at Shanghai Chest Hospital from January 2019 to May 2021 were included. The primary outcome was the incidence of hypoxemia during OLV between VDLT and cDLT. Secondary outcomes included bronchoscopy use, the degree of PaO_2_ decline, and arterial blood gas indices.

**Results:**

A total of 1,780 patients were finally analyzed in propensity score-matched cohorts (VDLT vs. cDLT 1:1 *n* = 890). The incidence of hypoxemia decreased from 6.5% (58/890) in cDLT group to 3.6% (32/890) in VDLT group (Relative Risk [RR]: 1.812, 95% CI: 1.19–2.76, *p* = 0.005). The use of bronchoscopy was reduced by 90% in VDLT group (VDLT 10.0% (89/890) vs. cDLT 100% (890/890), *p* < 0.001). PaO_2_ after OLV was 221 [136.0–325.0] mmHg in cDLT group compared to 234 [159.7–336.2] mmHg in VDLT group, *p* = 0.003. The percentage of PaO_2_ decline was 41.4 [15.4–61.9] % in cDLT group, while it was 37.7 [8.7–55.9] % in the VDLT group, *p* < 0.001. In patients who suffered from hypoxemia, there were no significant differences in arterial blood gas indices or the percentage of PaO_2_ decline.

**Conclusion:**

VDLTs reduce the incidence of hypoxemia and the use of bronchoscopy during OLV compared with cDLTs. VDLT may be a feasible option for thoracoscopic surgery.

## Introduction

Hypoxemia during OLV may jeopardize patient's safety and pose a challenge to the anesthesiologist and surgeon. It was reported that about 9%–28% of patients undergoing OLV during thoracic surgery developed severe arterial hypoxemia (PaO_2_ < 60–80 mmHg) (1). Several factors increase the chance of severe hypoxemia during OLV: morbid obesity, surgery on the right lung (which is larger than the left), patients who have had previous contralateral lung surgery, a high percentage of ventilation or perfusion to the operative lung on preoperative V/Q scan and hypoxic pulmonary vasoconstriction (HPV) is attenuated (2). Displacement of the endobronchial double-lumen tube (DLT) is thought to be one of the leading anesthesia-related causes that can be corrected (1). Fiberoptic bronchoscopy (FOB) has been recommended as the gold standard for verification of the position of the conventional DLT (cDLT) during OLV (3, 4). However, FOB does not provide continuous visualization of the tube's position. Multiple studies have revealed a high incidence of cDLT malposition (35%–48%), even when inserted by experienced providers (5, 6).

**Figure 1:**
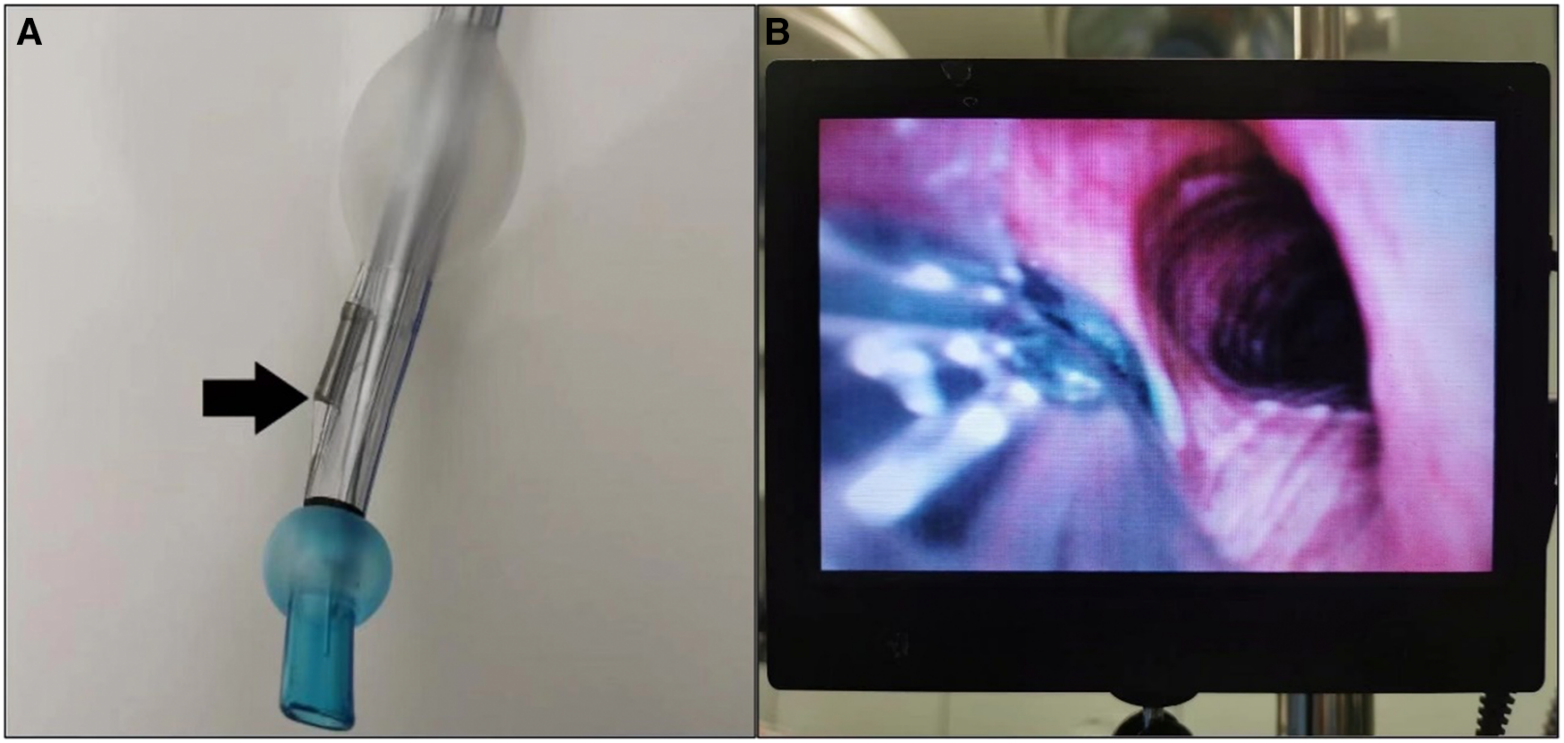
(**A**) Video-assisted DLT (well lead medical Co. Ltd.). The arrow indicates the optical sensor, which is licated at the junction of the tracheal and bronchial lumens. (**B**) An external display connected with VDLT, which can continuously dis-play the position of the tube in the airway.

VDLT (Well Lead Medical Co., Ltd. Guangzhou, China) (Figure 1) is a new type of DLT with an integrated high-resolution camera embedded at the distal end of the tracheal lumen, allowing continuous visualization of the trachea and carina during placement and throughout the entire surgical procedure (7–9). This device has been depicted to reduce the time required to intubate and confirm tube placement after patient surgical positioning (10).

Unlike cDLT, VDLT provides continuous surveillance throughout the surgical procedure, allowing for the early identification and treatment of tube displacement (3). Whether it may reduce the incidence of hypoxemia during OLV remains unknown. Our hospital is one of China's largest specialized thoracic hospitals, performing approximately 21,000 OLV surgeries annually. We hope to gain valuable evidence for the clinical use of VDLT through real-world clinical data by determining whether it helps to reduce hypoxemia.

## Materials and methods

The study was approved by the Institutional Review Board (IRB) of Shanghai Chest Hospital, Shanghai Jiao Tong University (Ref. No. IS2179, Chairman Ning Zheng). A waiver of informed consent was granted due to the retrospective nature of the study.

This retrospective cohort study was conducted at Shanghai Chest Hospital, Shanghai, China. We entered the data of every patient who underwent thoracoscopic surgery at Shanghai Chest Hospital, Shanghai Jiao Tong University from the Anesthesia Information System Database (Beijing Easy Monitor Technology Co., Ltd.).

Anesthesia procedure: After 3 min of anesthesia induction, a DLT was inserted with a visual laryngoscope. According to the type of DLT used, the patients were divided into two groups: VDLT (Well Lead Medical Co. Ltd.) and cDLT (Shiley™, Covidien Ireland Limited; or Portex, Smiths Medical International, Ltd.). Anesthesiologists can freely choose the types of DLT based on their experience. VDLT group can provide continuous visualization of the trachea and carina during placement throughout the entire surgical procedure. cDLT group used FOB to determine the position before and after OLV. We adjusted the breathing parameters after connecting the anesthesia machine as follows: tidal volume (VT) 8–10 ml/kg and respiration rate (RR) 10–12 times/min in volume-controlled ventilation (VCV) mode. VT 6–8 ml/kg and RR 12–14 times/min in OLV and an inhaled oxygen concentration of 100% during surgery. Invasive arterial pressure monitoring and deep venous catheterization were used. Arterial blood gas was measured before and after OLV, as appropriate. In our institution, the first blood gas during OLV was obtained routinely 15–30 min after OLV.

Adult patients who underwent elective thoracoscopic lung resection surgery and required VDLTs or cDLTs for OLV at Shanghai Chest Hospital from January 2019 to May 2021 were included. Data collection included age, gender, height, weight, body mass index (BMI), ASA physical status, surgical side, surgery approach, type of surgery, type of anesthesia, surgical duration, anesthesia duration, arterial blood gas indices before OLV and 30 min after OLV (OLV 30 min), and FOB use.

Inclusion criteria: age ≥18 years, ASA physical status I–III, elective thoracoscopic lung resection surgery, DLT used during OLV, and arterial blood gas indices (pH, PaO_2_, PaCO_2_, Hct, BE, and Lac) before OLV and 30 min after OLV. Exclusion criteria: missing data, outliers, bilateral lung surgery, PaO_2 _< 80 mmHg before OLV, and previous contralateral lung surgery. According to the type of DLT used, the patients were divided into two groups: VDLT and cDLT.

**Figure 2:**
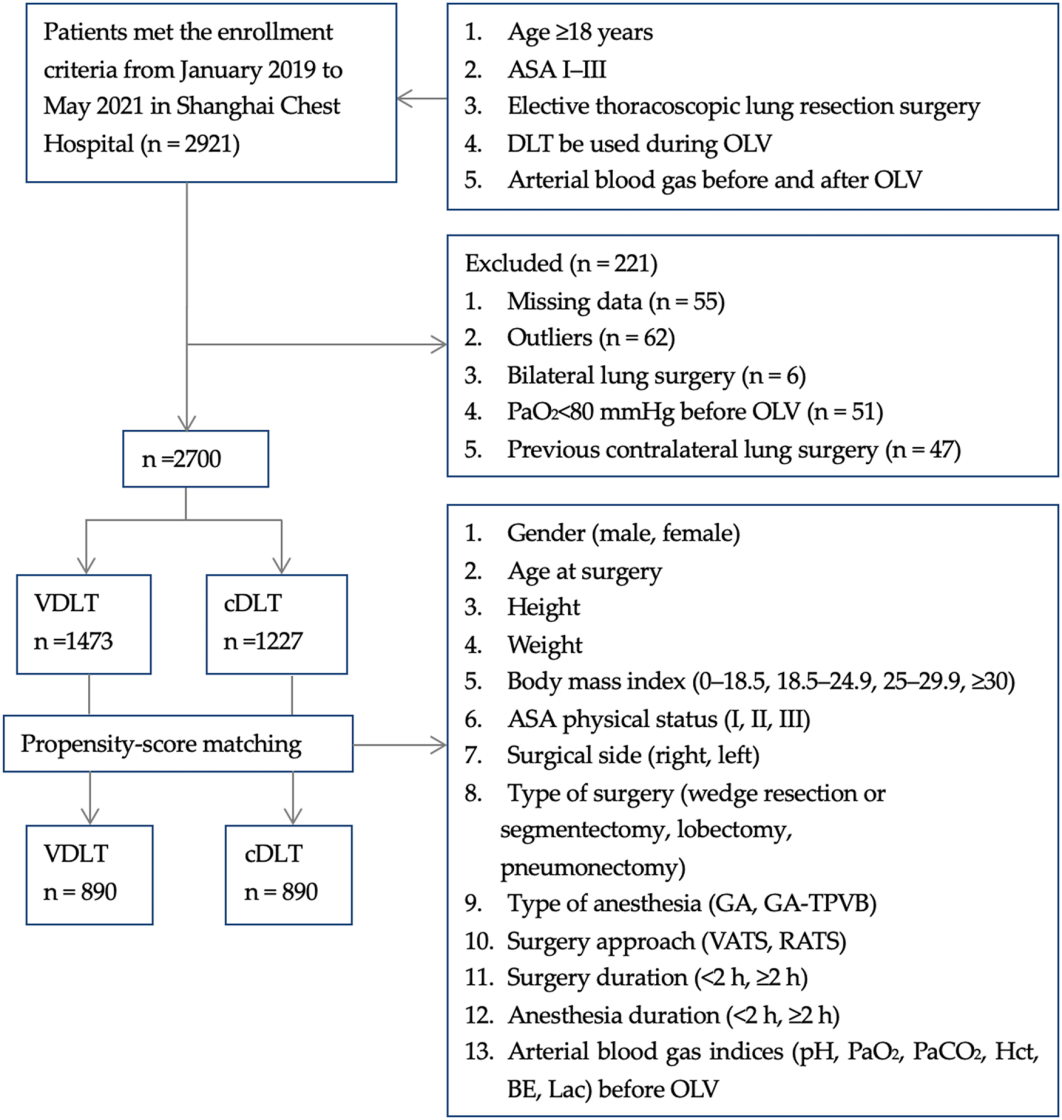
Flow chart for the inclusion and exclusion of patients.

To minimize potential selection bias, propensity score matching (PSM) was performed to balance the confounding factors between the two groups. We used a 1:1 ratio within a caliper width of 0.2 of the standard deviation of the logit of the propensity score (11, 12). Patients were matched based on the following variables: gender (male or female), age at surgery, height, weight, BMI (0–18.5, 18.5–24.9, 25–29.9, ≥30 kg/m^2^), ASA physical status (I, II, III), surgical side (right or left), type of surgery (wedge resection or segmentectomy, lobectomy, and pneumonectomy), type of anesthesia (general anesthesia [GA], general anesthesia-thoracic paravertebral blockade [GA-TPVB]), surgery approach (video-assisted thoracic surgery [VATS], robot-assisted thoracoscopic surgery [RATS]), surgery duration (<2 h, ≥2 h), anesthesia duration (<2 h, ≥2 h), and arterial blood gas indices (pH, PaO_2_, PaCO_2_, Hct, BE, and Lac) before OLV. The study flow chart is displayed in Figure 2.

The primary outcome was the incidence of hypoxemia during OLV. Hypoxemia was defined as PaO_2 _< 80 mmHg with an FIO_2_ of 100%. Hypoxemia during OLV was treated by the following strategies (13): First, 5 cm H_2_O of PEEP was applied to the dependent lung. Second, continuous positive airway pressure (CPAP) of 2–5 cm H_2_O of continuous positive airway pressure (CPAP) was applied to the non-dependent lung. Finally, brief periods of two lung ventilation were used.

Secondary outcomes: The rate of FOB use during OLV, the percentage of PaO_2_ decline [(PaO_2 before OLV_-PaO_2 OLV 30 min_)/PaO_2 before OLV_], and in patients with hypoxemia arterial blood gas indices (pH, PaO_2_, PaCO_2_, Hct, BE, and Lac) after OLV 30 min.

In this case, it was expressed as mean ± standard deviation (m ± SD), and if it did not follow a normal distribution, it was described as the median ± quartile interval (m ± QR). The count data were expressed as constituent ratios or percentages. The nonparametric rank-sum test was used for the non-normal distribution, and the chi-square test was used for the rate comparison. *p* < 0.05 was considered to indicate statistically significant. Absolute standardized difference (ASD) was used for covariates’ balance assessments and ASD < 0.1 can be considered as a sign of balance. All analyses were conducted using a computerized statistical software program (IBM SPSS Statistics, Version 26.0, IBM Corp., Armonk, NY, USA). Graphpad 7.0 was used to draw the graphics.

## Results

In this study, 2,921 patients were screened for the eligibility criteria, and 221 patients were excluded according to the exclusion criteria. Finally, 2,700 patients (1,473 in the VDLT group and 1,227 in the cDLT group) were enrolled in the pre-PSM study cohort. After calculating the propensity scores, 890 pairs were matched (Figure 2).

Detailed characteristics of the patients are listed in Table 1. No significant differences were found between the two groups in terms of age at the time of surgery, weight, BMI, ASA physical status (I, II, III), surgery duration (<2 h, ≥2 h), anesthesia duration (<2 h, ≥2 h), and blood gas indices (PaO_2_, PaCO_2_, pH, BE, Hct, and Lac) before OLV.

**Table 1:** VDLT vs. cDLT: baseline clinical characteristics (before and after PSM matching).

Patient characteristics	Before PSM matching				After PSM matching			
	VDLT (*n* = 1,473)	cDLT (*n* = 1,227)	*p*-value	ASD	VDLT (*n* = 890)	cDLT (*n* = 890)	*p*-value	ASD
Male gender, *n* (%)	782 (53.1%)	582 (47.4%)	0.003*	0.113^#^	448 (50.3%)	441 (49.6%)	0.740	0.016
Age at surgery (year), median [quartile]	63 [55–69]	63 [55–68]	0.170	0.053	63 [55–69]	63 [54–69]	0.851	0.013
Height (cm), median [quartile]	165 [160–170]	165 [159–170]	0.017*	0.092	165 [160–170]	165 [159–170]	0.900	0.008
Weight (kg), median [quartile]	63 [57–71]	63 [56–70]	0.059	0.073	63 [57–71]	64 [56–70]	0.628	0.033
BMI (kg/m^2^), *n* (%)			0.347	0.036			0.501	0.023
0–18.5	49 (3.3%)	57 (4.6%)			32 (3.6%)	45 (5.1%)		
18.5–24.9	985 (66.9%)	801 (65.3%)			589 (66.2%)	576 (64.7%)		
25–29.9	395 (26.8%)	334 (27.2%)			243 (27.3%)	242 (27.2%)		
≥30	44 (3.0%)	35 (2.9%)			26 (2.9%)	27 (3.0%)		
ASA physical status, *n* (%)			0.604	0.020			0.459	0.029
I	46 (3.1%)	47 (3.8%)			24 (2.7%)	29 (3.3%)		
II	1,097 (74.5%)	908 (74.0%)			674 (75.7%)	652 (73.3%)		
III	330 (22.4%)	272 (22.2%)			192 (21.6%)	209 (23.5%)		
Surgical side, *n* (%)			<0.001*	0.713^#^			0.497	0.032
Right	1,123 (76.2%)	534 (43.5%)			545 (61.2%)	531 (59.7%)		
Left	350 (23.8%)	693 (56.5%)			345 (38.8%)	359 (40.3%)		
Type of surgery, *n* (%)			<0.001*	0.068			0.365	0.007
Wedge resection/segmentectomy	363 (24.6%)	359 (29.3%)			242 (27.2%)	252 (28.3%)		
Lobectomy	1,095 (74.3%)	838 (68.3%)			636 (71.5%)	619 (69.6%)		
Pneumonectomy	15 (1.0%)	30 (2.4%)			12 (1.3%)	19 (2.1%)		
Type of anesthesia, *n* (%)			<0.001*	0.234^#^			0.326	0.047
GA	859 (58.3%)	853 (69.5%)			550 (61.8%)	570 (64.0%)		
GA–TPVB	614 (41.7%)	374 (30.5%)			340 (38.2%)	320 (36.0%)		
Surgery approach, *n* (%)			<0.001*	0.156^#^			0.861	0.008
VATS	1,411 (95.8%)	1,208 (98.5%)			873 (98.1%)	874 (98.2%)		
RATS	62 (4.2%)	19 (1.5%)			17 (1.9%)	16 (1.8%)		
Surgery duration (h), *n* (%)			0.791	0.010			0.598	0.025
0–2 h	636 (43.2%)	536 (43.7%)			383 (43.0%)	372 (41.8%)		
≥2 h	837 (56.8%)	691 (56.3%)			507 (57.0%)	518 (58.2%)		
Anesthesia duration (h), *n* (%)			0.515	0.025			0.558	0.028
0–2 h	185 (12.6%)	144 (11.7%)			110 (12.4%)	102 (11.5%)		
≥2 h	1,288 (87.4%)	1,083 (88.3%)			780 (87.6%)	788 (88.5%)		
Blood gas before OLV								
PH, median [quartile]	7.38 [7.35–7.40]	7.38 [7.35–7.40]	0.714	0.014	7.38 [7.35–7.40]	7.38 [7.35–7.40]	0.830	0.014
PaO_2_ (mmHg), median [quartile]	391 [322–441]	391 [328–444]	0.571	0.022	388 [322–440]	394 [328–449]	0.150	0.096
PaCO_2_ (mmHg), median [quartile]	40.4 [37.1–43.7]	40.6 [37.1–44.0]	0.294	0.041	40.4 [37.1–43.9]	40.9 [37.3–44.1]	0.217	0.083
Hct (%), median [quartile]	41.6 [37.5–45.7]	41.2 [37.4–45.0]	0.213	0.048	45.5 [41.2–49.2]	45.7 [41.9–49.6]	0.280	0.073
BE (mmol/L), median [quartile]	−1.2 [−2.3 to −0.1]	−1.0 [−2.1 to −0.1]	0.075	0.069	−1.2 [−2.1–0.0]	−1.0 [−2.1 to −0.1]	0.140	0.099
Lac (mmol/L), median [quartile]	1.2 [0.9–1.5]	1.1 [0.9–1.4]	0.273	0.042	1.2 [0.9–1.5]	1.2 [0.9–1.5]	0.582	0.037

VDLT, video double-lumen tube; cDLT, conventional double-lumen tube; PSM, propensity-matched analysis; ASD, absolute standardized difference; BMI, body mass index; ASA, American Society of Anesthesiologists; GA, general anesthesia; TPVB, thoracic paravertebral blockade; VATS, video-assisted thoracic surgery; RATS, robot-assisted thoracoscopic surgery.

* Indicates a statistical difference between the two groups.

^#^ Indicates a ASD value that is greater than 0.1.

The PSM identified 890 cases in each group. There was no difference in the baseline data between the two groups after PSM which was indicated by the ASD value (Table 1). In the matched cohort, there were significant differences in outcomes between the VDLT and cDLT groups.

The incidence of hypoxemia decreased from 6.5% (58/890) in cDLT group to 3.6% (32/890) in VDLT group (Relative risk [RR]: 1.812, 95% CI: 1.19–2.76, *p* = 0.005). The use of FOB was reduced by 90% in VDLT group (VDLT 10.0% (89/890) vs. cDLT 100% (890/890), *p* < 0.001). PaO_2_ after OLV was 221 [136.0–325.0] mmHg in cDLT group compared to 234 [159.7–336.2] mmHg in VDLT group, *p* = 0.003. The percentage decrease in PaO_2_ was 41.4 [15.4–61.9] % in cDLT group and 37.7 [8.7–55.9] % in VDLT group, with *p* < 0.001. Patients in the VDLT group had lower pH, Hct, and BE levels than those in the cDLT group, but neither group exceeded normal levels (Table 2).

**Table 2:** VDLT vs. cDLT: outcomes (before and after PSM matching).

Outcomes	Before PSM matching			After PSM matching		
	VDLT (*n* = 1,473)	cDLT (*n* = 1,227)	*p*-value	VDLT (*n* = 890)	cDLT (*n* = 890)	*p*-value
Hypoxemia, *n* (%)	64 (4.3%)	75 (6.1%)	0.038*	32 (3.6%)	58 (6.5%)	0.005*
FOB used, *n* (%)	142 (9.6%)	1,227 (100%)	<0.001*	89 (10.0%)	890 (100%)	<0.001*
Blood gas after OLV						
pH, median [quartile]	7.36 [7.33–7.38]	7.36 [7.33–7.39]	<0.001*	7.36 [7.33–7.38]	7.36[7.33–7.39]	0.002*
PaO_2_ (mmHg), median [quartile]	220 [156–318.0]	229 [141–327]	0.948	234 [159.7–336.2]	221[136–325]	0.003*
PaCO_2_ (mmHg), median [quartile]	41.8 [38.4–46.2]	41.0 [37.6–45.4]	0.002*	41.8 [38.4–46.5]	41.4[37.8–45.6]	0.053
Hct (%), median [quartile]	39.1 [35.3–43.1]	39.1 [35.4–42.9]	0.910	38.9 [35.2–42.7]	39.8[36.3–43.4]	0.002*
BE (mmol/L), median [quartile]	−1.9 [−3.1 to −0.8]	−1.7 [−2.8 to −0.7]	0.005*	−1.8 [−3.0 to −0.8]	−1.6[−2.8 to −0.6]	0.044*
Lac (mmol/L), median [quartile]	0.8 [0.6–1.1]	0.80 [0.6–1.1]	0.016*	0.8 [0.6–1.1]	0.8[0.6–1.1]	0.323
PaO_2_ decline (%), median [quartiles]	40.6 [14.9–58.2]	39.5 [14.8–59.8]	0.699	37.7 [8.7–55.9]	41.4[15.4–61.9]	<0.001*

FOB, fiberoptic bronchoscopy.

* Indicates a statistical difference between the two groups.

**Figure 3:**
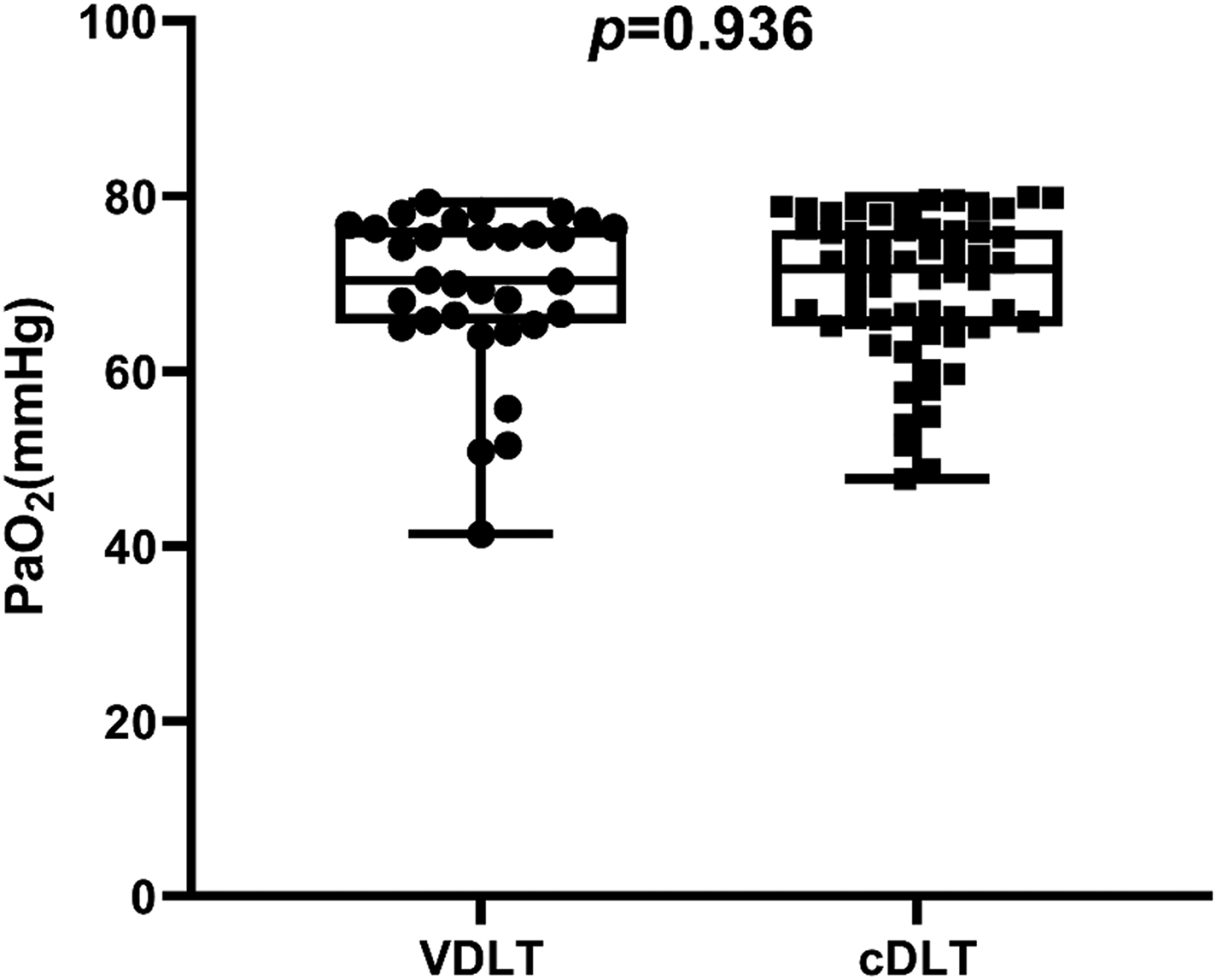
The margin of PaO_2_ after OLV in patients with hypoxemia. The centerlines show the medians; the box limits indicate the 25th and 75th percentiles; whiskers extend to data points that are less than 1.5 × interquartile ratios away from the first/third quartile.

There was no significant difference in arterial blood gas indices or the percentage of PaO_2_ decline in hypoxemic patients (Table 3). In VDLT group, the PaO_2_ median level was 70.4 [65.4–76.4] mmHg, with extreme values of 41.4 and 79.3 mmHg. In cDLT group, the PaO_2_ median level was 71.7 [65.2–76.1] mmHg, with extreme values of 47.7 and 79.9 mmHg (Figure 3).

**Table 3:** VDLT vs. cDLT: outcomes in patients with hypoxemia (after PSM matching).

Outcomes in patients with hypoxemia	After PSM matching		
	VDLT (*n* = 32)	cDLT (*n* = 58)	*p*-value
Arterial blood gas after OLV			
pH, mean ± SD	7.33 ± 0.05	7.34 ± 0.05	0.511
PaO_2_ (mmHg), median [quartile]	70.4 [65.4–76.4]	71.7 [65.2–76.1]	0.936
PaCO_2_ (mmHg), mean ± SD	45.8 ± 5.9	46.3 ± 6.3	0.709
Hct (%), mean ± SD	39.2 ± 6.98	41.3 ± 5.71	0.119
BE (mmol/L), mean ± SD	−2.2 ± 1.5	−1.67 ± 1.7	0.449
Lac (mmol/L), median [quartile]	0.8 [0.6–1.1]	0.8 [0.6–1.2]	0.889
PaO_2_ decline (%), median [quartiles]	77.6 [70.7–80.5]	76.3 [70.3–81.6]	0.960

## Discussion

We conducted a large retrospective cohort study comparing the use of VDLTs versus cDLTs in elective thoracoscopic lung resection surgery. This study confirmed our hypothesis that the incidence of hypoxemia during OLV is decreased by using VDLTs compared to cDLTs.

Several factors contribute to hypoxemia during OLV, with malposition of the DLT being one of the primary causes that can be corrected (1). In our study, the incidence of hypoxemia decreased from 6.5% (58/890) in cDLT group to 3.6% (32/890) in VDLT group (RR: 1.812, 95% CI: 1.19–2.76, *p* = 0.005). Similarly, a retrospective analysis of 1,170 patients undergoing OLV revealed that most of the cases required a correction of tube position to alleviate hypoxemia (14). Because of the inherent difficulties associated with the use of DLT, inadequate monitoring of the tube may result in hypoxemia. Furthermore, cDLTs malposition can repeatedly occur despite initial adjustment with a FOB (15). In contrast, VDLTs provide a promising solution with the embedded camera (16). VDLTs enable anesthesiologists to be forewarned of the loss of lung isolation by directly visualizing the dislodgement of the endobronchial cuff. Real-time visualization enables a rapid diagnosis and correction of the DLT malposition in the lateral position without potentially disrupting ventilation. VDLTs probably decrease the incidence of hypoxemia during OLV by taking these factors together.

At the beginning of thoracic surgery, switching mechanical ventilation from two-lung ventilation to OLV always results in declining PaO_2_ levels (30%–50%); consequently, reduced surface area available for gas exchange and variable intrapulmonary shunting (17). In our study, at OLV 30 min, PaO_2_ levels decreased by about 40% (VDLT 37.7 [8.7–55.9] % vs. cDLT 41.4 [15.4–61.9] %, *p* < 0.001). As we know, HPV progressively increases and reaches its maximal state at 60–120 min (18). In our study, the arterial blood gas of OLV was measured about 30 min after OLV. Therefore, the influence of HPV can be considered no different. Other factors, such as morbid obesity, right lung surgery, previous contralateral lung surgery, and a high percentage of ventilation or perfusion to the operative lung on preoperative V/Q scan, increase the risk of hypoxemia during OLV (2). To minimize the potential selection bias, PSM was performed to balance these confounding factors between the two groups. There was no difference in the baseline data between the two groups after PSM (Table 1).

The rate of FOB uses significantly decreased when intubated with a VDLT (from 100% to 10.0%), which is consistent with other studies (7, 19). However, the price of a VDLT is relatively higher than that of a cDLT. Accounting for the initial purchase, sterilization, maintenance, and repair, using a FOB can also be expensive. According to an earlier cost-effectiveness analysis comparing VDLT (VivaSight-DL) and cDLT in thoracic surgery, VDLT can result in significant cost savings (VDLT $US299.96 vs. cDLT $US347.61 per procedure) by reducing the use of FOB (19). In another study, the researchers found that using VDLT is economical, whether using reusable or disposable FOB to verify that the cDLT is positioned correctly. Moreover, using FOB can potentially interrupt ventilation on its own, which may cause hypoxemia and hypercarbia. Using FOB to check and adjust the position of cDLTs is time-consuming and inconvenient, especially in RATS. There is a limitation of the VDLT due to secretions that prevent an adequate view of the carina and correct one-lung ventilation (16, 20, 21). In our study, 10.0% (89/890) of VDLT required the use of FOB, mainly due to poor image field of view and the need for subcarinal bronchial examination. Therefore, VDLT cannot completely replace FOB at present.

It should be noted that the patients with hypoxemia PaO_2_ median level was 70.4 [65.4–76.4] mmHg, with extreme values of 41.4 and 79.3 mmHg in the VDLT group. In the cDLT group, the median PaO_2_ level for patients with hypoxemia was 71.7 [65.2–76.1] mmHg, with extreme values of 47.7 and 79.9 mmHg. In some cases of severe hypoxemia, the underlying cause may be the patient's pathophysiological condition, such as HPV impairment, rather than the DLT displacement. Additionally, the study found differences in pH, Hct, and BE between the two groups, but neither group exceeded normal levels for these parameters. There may not be a clinically significant difference between the indices.

This study has several limitations. As with any retrospective study, it was impossible to control for bias and confounding factors because there was no randomization or blinding. It should be appreciated that this study is from a center with a very high volume of thoracic surgery, and the anesthesiologists involved have extensive experience in the use of VDLT. Therefore, the average learning curve of a novice anesthesiologist using VDLT cannot be predicted well. In the meanwhile, different intraoperative drugs, such as inhalation versus intravenous anesthetics, might affect oxygenation specifically by influencing hypoxic pulmonary vasoconstriction (HPV). Accurate acquisition of intraoperative drug dosage is impractical due to the objectivity of a retrospective study. Notably, our center routinely administers total intravenous anesthesia to all patients, so there is little difference between the groups in terms of intraoperative drugs. Moreover, factors such as anatomical tracheal deviations, selection of tube size and tube malpositioning which may influence hypoxemia were not included in this study since they do not affect the selection of endotracheal tube in our grouping, but it's important to consider the factors which may influence the outcome as complete as possible. In addition, this study did not follow up on the long-term prognoses of patients. Whether using VDLT can reduce the incidence of post-intubation complications, postoperative hypoxemia or postoperative pulmonary complications requires further research.

## Conclusion

In a propensity score-matched analysis, we found that VDLTs can reduce the incidence of hypoxemia during OLV compared with cDLTs. Additionally, the use of FOB decreased significantly with a VDLT compared to a cDLT. Therefore, continuous visualization with a VDLT appears beneficial in clinical practice.

## Data Availability

The raw data supporting the conclusions of this article will be made available by the authors, without undue reservation.
